# American Dental Association

**Published:** 1892-01

**Authors:** 


### AMERICAN DENTAL ASSOCIATION.

*August 5, 1891.—Second Day.—Evening Session.*

MEETING called to order at 8.40, with the President in the chair.

*Dr. Walker.*—As there are many dentists here from all over the country, many of them have requested me to ask if at this time it would be proper to have a report made in regard to the condition of the legislation that has recently occurred between the International Tooth Crown Company and the Dental Protective Association. I thought I would bring the matter before the Association to see if it would be proper to have a report made, and I move that the chairman be given five minutes in which to make a statement concerning that litigation.

This was adopted.

*Dr. Crouse.*—The case was lost. We got the unanimous vote of the Supreme Court in our favor. We have a suit pending which is to be a test suit on the Low Bridge patent. We have assumed the control of it, and it will be a test suit. It is not ended; it will probably be the most important we have had. We feel sure of winning this one. We were not sure of winning the other. I believe that when our testimony is all in, so the other side can see what we have in our possession relative to the patent, that they will never bother us again. We have plenty of evidence that makes us sure that we will beat them in the higher court, and we think we can convince them that we are right before they go to court.

The Low Bridge includes the banded crown with a tooth attached. It is a kind of dummy on a gold crown. You will see when the circular comes. We will have cuts illustrating, so that every one can see how important it is that we should win the suit and have all the evidence in our favor that we possibly can.

Dr. Shepard, on behalf of the committee appointed to examine into the affairs of the Dental Protective Association of the United States, reports that they have examined the books and accounts, and find them kept in a business-like manner; the affairs have been managed very economically; all bills have been paid, and the surplus is deposited in bank to the credit of the Association.

Dr. Barrett, chairman of the committee on the President's annual address, reported that the committee recommended several changes in the constitution, which were read by him.

Dr. McKellops stated a case in which a rubber-dam clamp had been swallowed by a patient, and requested Dr. Peirce to report the case.

*Dr. Peirce.*—Mr. President, Dr. Shepard has called on me to report what I stated to him. A week ago a dentist in Philadelphia called on me with his patient. He had in his hand a rubber-dam clamp of the largest size, and he said his patient had swallowed a clamp of that kind, and asked me what to do. I told him to go to Dr. J. Solis-Cohen, about four doors above me. They left my office. On the day before I left Philadelphia I met Dr. Cohen, and asked him about the matter. He said, "I took hold of the clamp with my forceps, but I found that I was so lacerating the œsophagus that I would irreparably hurt the patient. I told him to go home and live on mashed potatoes for two days. Yesterday the patient brought me the clamp, which had been passed without pain. When anything is swallowed into the œsophagus, I direct my patient to exist on mashed potatoes for a while, and that invariably passes the obstruction into the stomach, then into the alimentary canal, and so ends the trouble."

*Dr. Brophy.*—I believe the paper of Dr. Barrett calls for some discussion. I think some of the members of the Association who were not here at the time the paper was read may desire to say something. Some may not have been able to discuss it when the paper was read.

No one desiring to speak, the subject was passed.

Dr. John S. Marshall, of Chicago, read a paper on the use of pyoktanin in the treatment of cancerous growths.

### PYOKTANIN BLUE IN THE TREATMENT OF CANCEROUS GROWTHS, WITH ONE CASE IN ILLUSTRATION.

BY JOHN S. MARSHALL, M.D.[1]

Mr. President and Gentlemen,—I desire to call your attention to the new antiseptic drug pyoktanin and its use in the treatment of cancerous growths.

Pyoktanin was discovered by Professor J. Stilling, of Strasburg, Germany. It is one of the aniline compounds and a powerful antiseptic.

There are two varieties of the drug, one of a yellow color and

---

[1] Oral Surgeon to St. Luke's Free Hospital and Mercy Hospital, Chicago, Illinois.

the other a deep blue; each produces a corresponding deep stain. The blue dye has greater antiseptic powers than the yellow, and is generally given the preference when the deep purple color imparted to the tissues by its use is not objectionable.

Professor Stilling and Dr. Wortmann, after making careful and elaborate chemical and bacteriological research, claim for pyoktanin as an antiseptic,—

"*First.* It has no injurious effects upon the human organism.

"*Second.* It is absolutely destructive to all bacteria or other micro-organisms absorbing it.

"*Third.* It rapidly permeates and diffuses through both healthy and diseased tissues and fluids, and penetrates the enveloping membranes and internal matter of all bacterial colonies harbored by them."

For antiseptic purposes it is used in aqueous solution, the strength ranging from 1 in 1000 to 1 in 100, the parts being irrigated or painted with the solution. The only objection to its use by these methods is the discoloration imparted to the surrounding tissues and the staining of the clothing; these stains, however, are easily removed by washing in alcohol.

Dr. H. J. Bolt, of New York,[1] speaks very highly of pyoktanin blue as an antiseptic in connection with the treatment of various suppurative and purulent conditions coming under the notice of the gynecologist.

Dr. Roswell Park, of Buffalo, says (in "Annals of Surgery") that pyoktanin cannot be relied upon in surgical practice except in such strength as to make it dangerous. In gonorrhœa it is disappointing. Upon granulating surfaces it exerts a desirable effect, but is no more valuable than other substances as easy of access, while the stain is often objectionable. In ophthalmological practice it has not come up to the requirements of the day. It has few qualities not possessed by numerous other drugs of its class, and on the whole it is disappointing.

For a few weeks only I have been testing its value as an antiseptic in the treatment of phagedenic pericementitis, but am not prepared to give the results of these experiments at this time, as it is my intention to present them to the profession in the form of a separate paper. This much, however, I may say in regard to it, that it gives promise of being a valuable addition to our yet very meagre armamentarium for combating the effects of this disease.

---

[1] Journal American Medical Association, July 11, 1891, p. 76.

The field, however, in which at present it seems to give the greatest promise of usefulness is in cases of cancerous growths of all varieties which have passed beyond the hope of successful removal with the cautery or the knife, by mitigating the sufferings and prolonging the lives of these unfortunate individuals. Although no positive cures have yet been announced as resulting from its use, many have been greatly benefited and some few have been so far improved as to hold out the hope of a positive cure.

In an article by Dr. Willy Meyer, of the German and New York Skin and Cancer Hospitals, which appeared in the *Medical Record* of April 25, 1891, will be found a *résumé* of the opinions of the most eminent German surgeons upon its use and value in the treatment of cancerous growths, with the cases upon which it had been used and the results so far obtained, together with histories of four cases treated by himself.

Let me quote a few paragraphs from this most interesting paper.

"Stilling mentions in his second paper that he succeeded in curing with pyoktanin an extensive exulcerated epithelioma of the face (ulcus rodens) which, beginning in the middle of the dorsum of the nose, involved the entire wall of the lachrymal sac and half of the upper eyelid.

"On January 30, 1891, Professor von Mosetig-Moorhof read a very interesting paper before the Vienna Society of Physicians 'A Contribution to the Treatment of Inoperable Malignant Growths,' in which he reported on his experience with pyoktanin in such cases.

"Stilling was the first to suggest parenchymatous injections of aniline dyes in inoperable neoplasms, and advised the use of such dyes as will dissolve slowly in a six-tenths-per-cent. solution of chloride of sodium,—viz., blood. He recommends this precaution to guard against the too rapid diffusion of the injected fluid through the tissues and the possibility of the dye entering the blood, as he feared the absorption of too large a quantity by the red corpuscles might possibly destroy their function and thus endanger life. He is of the opinion that the blue dye is best for parenchymatous injection, as it is the most powerful antiseptic of this class of drugs and is soluble in only seven-tenths to eight-tenths part in 1000 of water.

"With regard to its practical application to cancerous growths, he suggests the employment of Nussbaum's method of treatment first, which is to tie the principal arteries which supply the neoplasm to arrest its growth, and then to inject the tumor in all its parts with a solution of pyoktanin blue.

"Von Mosetig presented four of his cases, previously referred to, at a recent meeting of the Vienna Society of Physicians (March 13). The patients had all been materially improved, but were not yet cured. Von Mosetig concludes that we had better be careful with this treatment in cases of malignant growths which have developed in the neighborhood and around large blood-vessels. He is inclined to avoid injection into the tumors which involve large veins, and also into those which cannot easily be reached by the needle of the syringe. He now prefers a solution of 1 to 500 as a standard solution, which is filtered through heated asbestos to remove undissolved crystals, which might plug the needle, and makes the injections in a concentric manner, letting his needle enter into the surrounding healthy tissues. In several hundreds of these injections neither he nor his assistants have ever seen any untoward accident. Microscopical examination of extirpated pieces of three different new growths, which had been injected, made by Riehl, of Vienna, demonstrated that the nerves, muscles, connective tissues, and young epithelial cells were diffusely dyed, but the nuclei only slightly, or not at all.

"Billroth is rather sceptical in regard to the value of pyoktanin injections in cases of malignant growths. Three patients, having far-advanced sarcoma (posterior aspect of thigh, sternum, popliteal space), got worse; one of them died of sepsis. The tumors were softened and the covering skin was perforated. This occurrence can only be looked upon, according to Billroth, as unfortunate to the patient. The exulcerated growth troubles the patients much more and will render their condition more dangerous. Patients afflicted with exulcerated growths also did not improve. Billroth does not believe that pyoktanin, when injected, has any specific influence whatsoever, but that it is merely the water, which, forcibly pressed into the tissues, makes them swell and later unable to live. The researches are continued in his clinic."

Dr. Meyer reports having tested the value of the drug in nine cases at the hospitals and two cases in private practice. The histories only of the first four hospital cases are related in his paper.

It would be extremely interesting to review in detail the histories of these cases, did time and space permit; but it will be sufficient for our present purpose to say that the report shows that marked improvement followed the treatment in each case, the severity of the pain was greatly mitigated, the diseased tissue took on a more healthy appearance, and in every case the tendency to spread to adjoining tissue seemed to be arrested.

In summing up his experience with pyoktanin, Dr. Meyer says,—

"In reviewing these four histories, and also considering my experience with pyoktanin as well as ethyl pyoktanin in my private patients, I should like to state the following facts in regard to the treatment of inoperable malignant growths with these aniline dyes, as far as they have been exhibited up to date (April 8):

"The aniline dyes, applied on the surface of exulcerated growths, in ointment or powder (about 1 to 200) or in substance, have a strong analgesic effect.

"Parenchymatous injections of pyoktanin solution (1 to 300), repeated every second day till fifth day, the dose not exceeding one and one-half drachms, have in my cases proven to be innocuous to the general system.

"The symptoms following the injections are either general or local.

"The general symptoms, which can also follow the internal use of methyl blue (as recommended for inoperable malignant neoplasms by Dr. Max Einhorn of this city), are nausea or vomiting, weak and slow pulse, headaches, general malaise. These symptoms may come on the same day or the day following the injection; as a rule, they do not come at all; now and then there is a slight rise of temperature, usually subsiding within twenty-four to forty-eight hours.

"The local symptoms are,—

"1. Pain following the injection. It may be of long or short duration, slight or severe. It is more severe where the cancerous infiltration affected by the injection is dense (epithelioma of face, disseminated cancer of breast, etc.). It seems that local anæsthesia may follow the injection.

"2. Œdema, coming on either acutely, and then accompanied with slight redness (non-inflammatory) and pain on pressure, or rather in a subacute form. This serous transudation of the growth may be the first step to reabsorption. The slight fever, observed in a number of patients, soon following the injection, had then to be looked at as being an aseptic one, possibly due to this reabsorption.

"3. Breaking down of the injected tissue, with perforation of the skin, or scar, which latter is the result of the operation (aseptic necrosis). By using a strong solution (1 to 200) the necrobiosis seems to be more rapid in smaller nodules. It may be advisable to use a weaker solution (1 to 500, instead of 1 to 300), as we can thus dye a larger portion of the growth in one sitting without increasing the dose of pyoktanin. We then inject a larger quantity of solution and still need not apprehend general symptoms. Perhaps the weaker solution will not destroy the tissue so rapidly, and thus rather induce reabsorption than necrosis. The sinus or sinuses established by the breaking down of the tissues give exit to a thick, dark-blue fluid, which, by microscopical examination, proves to be not colored pus, but *débris* of the injected neoplasm. It is generally intermingled with shreds of necrosed tissue. We also have to prove yet whether operative interference in this stage will really benefit our patients. Perhaps nature gets slowly rid of the necrosed tissue without help. As it seems, the carcinoma tends more to necrobiosis and perforation during this treatment than the sarcoma. I personally could so far only subject cancer cases to the color cure. (Von Mosetig had his most striking results in patients suffering from sarcoma (see above). He maintains that the injections, made under antiseptic precautions into neoplasms, which are still covered by healthy skin, produce necrobiosis and fatty degeneration, which is not followed by perforation but by reabsorption. Billroth, on the other hand, saw rapid softening and perforation in two out of three patients who suffered from sarcoma and were treated with the injections.)

"4. Breaking down of the injected tissue (necrobiosis), with subsequent reabsorption (Cf. 3). This will probably be oftener seen if we stop the injections for some time as soon as spots in the growth have softened, and only start them again after these spots have shrunk (provided, of course, that they did not perforate). Small, hard nodules in the skin, as seen especially in the disseminated cancer (recurring cancer of breast), can entirely disappear in a very few days, even if the pyoktanin solution was injected into their base. Perhaps the rapid disappearance is not reabsorption, but only an illusion, which is caused by œdema of the immediate surrounding tissue. A number of the nodules reappear after a few weeks, sometimes rather multiplied. The attempt at dyeing these small nodules directly, by pushing the needle right into them, is frequently unsuccessful, even in regard to the microscopical appearance, and causes severe pain.

"An infiltration of hitherto healthy tissue with the neoplasm, in consequence of this treatment, has not been observed in a single case.

"In no case did the growth spread during the treatment.

"Applications of pyoktanin in substance on exulcerated tumors slowly remove the diseased tissue in the shape of dry gangrene. There is no suppuration under the eschar, on account of the strong antiseptic qualities of the dye, and consequently no cachexia. Pyoktanin is, however, no deodorizer. It is also no stypticum.

"It may be advisable to combine the parenchymatous injections and local application of pyoktanin with giving aniline dyes internally. Pyoktanin is, in my cases at least, not borne by the stomach. Methyl blue seems at present to be the most preferable."

The reading of Dr. Meyer's paper greatly interested me from the surgical stand-point, for it so happened that just at that time a private patient, Mr. H., American, aged forty-one, general health good, family history good, referred to me by Professor Arthur B. Freeman, was suffering from a very extensive malignant neoplasm (small, round-cell sarcoma) originating in the antrum of Highmore, involving the right superior maxilla and malar bones, etc.

An operation, with the assistance of Professor Freeman, had been made for the removal of the growth in the antrum, on April 2, 1891, but so rapid was the extension of the disease following this procedure, that the second one, with the assistance of Professor John Owen, was performed on May 12 following, when the entire right superior maxilla, malar, palate, and turbinated bones, with portions of the sphenoid, were removed. The first manifestations of the disease were discovered only six weeks before the first operation. Ten days afterwards, when the patient was allowed to leave the hospital for his home, there was forming over the right ramus of the inferior maxilla an extensive induration.

It was determined, by the consent of the patient, that if there should be positive indications of farther spreading of the disease, or recurrence, that we would immediately begin the use of pyoktanin by parenchymatous injection.

I was led to this determination by the hope held out in the results which had followed its use in cases of a similar nature reported by Von Mosetig-Moorhof.

*May* 28.—The induration over the right ramus had assumed the size and shape of a hen's egg somewhat flattened laterally, and was unmistakably a farther extension of the disease.

Parenchymatous injections of a solution of pyoktanin 1 to 500 in a six-tenths-per-cent. solution of chloride of sodium in distilled water were commenced at this date.

Twenty minims were injected into the substance of the growth at four different points, five minims at each point. The utmost care was used to insure perfect aseptic conditions of the external surface of the tumor, the needles and syringe, the hands of the operator and the nurse, and in the preparation of the solution itself.

The following day the axillary temperature showed an elevation of one degree (at 9 A.M. it was 98.4°, at 2 P.M. it was 99.4°), which fell to normal in the evening. No other general symptoms were manifested.

*May* 31.—Parenchymatous injection of pyoktanin twenty-five minims at five different locations, five minims at each place.

Temperature remained normal; tumor larger and feels very hard.

*June* 3.—Injected thirty-five minims divided between four locations, endeavoring to reach a new point at each time. No elevation of temperature; tumor increasing in size.

*June* 6.—Injected forty minims, as before. Temperature normal, no unpleasant symptoms, appetite good. Pain in the region of the temporo-maxillary articulation and sense of fulness following the injection.

Skin over the tumor is tinged slightly purple.

Tumor about the same size, perhaps a little larger, and very hard.

*June* 8.—Injected forty minims, as before. Pain quite severe after the injection, evidently from the pressure of the solution; this gradually subsided in half an hour. No elevation of temperature or other constitutional symptoms.

*June* 10.—Tumor seems to be somewhat smaller. Injected forty minims. Decided to double the intervals between the injections, as the tumor seems to be thoroughly dyed, and watch the results. Skin over tumor quite purple.

*June* 14.—Tumor has been slowly but certainly decreasing in size since the last date, and has softened very considerably. Injected sixty minims divided between six different points. Pain quite severe for a few minutes after the injection.

*June* 17.—Slight redness at upper part of tumor; looks as though the skin was about to rupture; fluctuation quite perceptible at this point. Painted this location with collodion.

Injected sixty minims in lower part of neoplasm, which caused considerable pain for a little time.

Tumor slowly decreasing in size, most noticeable at upper part.

*June* 19.—Tumor ruptured upon the inner side into the mouth, discharging a considerable quantity of dirty-looking purplish fluid, evidently necrotic broken-down tissue stained with pyoktanin. Tumor soft and much smaller. No injection.

*June* 21.—No injection. Tumor soft and greatly reduced in size and discharging same dirty-looking fluid. No evidences of suppuration.

*June* 26.—Neoplasm still decreasing in size; discharge has ceased. Patient feels so much better that he desires to return to business.

Small point of coarse granulations near the anterior border of the velum palate. Injected thirty minims in this location.

*July* 1. Neoplasm has nearly disappeared, and the patient has gone back to his business.

The coarse granulations referred to have disappeared; they were doubtless due to irritation from the packing used to close the cavity into the nose.

*July* 13. Patient reported to-day for examination. Neoplasm has entirely disappeared. Discovered another point of coarse granular-looking tissue at the bottom of the cavity; applied pyoktanin (1 to 300 of lanolin) upon a piece of absorbent cotton held in place by the packing above referred to.

*July* 16. The coarse granular tissue at bottom of cavity has disappeared. Patient cautioned not to irritate the surface of the cavity by undue pressure of the packing, and instructed to report at once if at any time there should be evidence of recurrence.

REMARKS.

In the exhibition of the drug in this case I endeavored to profit by the experience contained in the published reports of others.

From the fact that in all the cases reported by Dr. Meyer the drug was not borne well by the stomach, I refrained from administering it in this manner; and as there was no external ulcerated surface, the use of stick or powdered pyoktanin was not indicated.

I chose the 1 to 500 in six-tenths-per-cent. chloride of sodium solution as being the safer strength, beginning the injections with twenty minims, and gradually increasing them to one drachm for the same reason.

The possibility of septic infection was guarded against by strict antiseptic precautions, and the needle wounds were covered each time with collodion.

From the beginning to the end of the treatment there has not been the slightest evidence of constitutional disturbance from the use of the drug, save the elevation of temperature of one degree, which occurred on the day following the first injection, and which,

I am quite inclined to believe, was the result of anxiety as to the outcome of the treatment, rather than to the influence of the drug. Pyoktanin in this case has certainly proved very beneficial, and gives promise of establishing a complete cure. Time only will demonstrate whether this promise is to be fully realized.

One thought further: The impression has been gradually growing in the minds of not a few noted surgeons, both of Europe and America, that the clinical facts surrounding the location and progress of primary carcinoma, and possibly sarcoma, in all their forms, would indicate an entirely local origin of these neoplasms, and that they might be caused by the lodgement or ingress of a specific micro-organism entering through abraded surfaces and wounds. I therefore ask, May not the results so far obtained in the use of this powerful but innoxious antiseptic drug—pyoktanin—upon malignant neoplasms be a further indication that this may be the correct theory of the origin of these growths, and the line upon which further experimentation will give the greatest hope of success?

If pyoktanin does not prove to be permanently efficacious, may we not hope that some other drug will yet be discovered, through the combined efforts of the chemist and the bacteriologist, that will eventually conquer this dreadful and loathsome scourge of humanity?

